# Positive Regulation of Estrogen Receptor Alpha in Breast Tumorigenesis

**DOI:** 10.3390/cells10112966

**Published:** 2021-10-31

**Authors:** Lucas Porras, Houssam Ismail, Sylvie Mader

**Affiliations:** Institute for Research in Immunology and Cancer, Université de Montréal, Montreal, QC H3T 1J4, Canada; lucas.porras@umontreal.ca (L.P.); houssam.ismail@umontreal.ca (H.I.)

**Keywords:** breast cancer, estrogen receptor alpha, luminal breast cancer, mammary gland, *ESR1*, *FOXA1*, *GATA3*

## Abstract

Estrogen receptor alpha (ERα, NR3A1) contributes through its expression in different tissues to a spectrum of physiological processes, including reproductive system development and physiology, bone mass maintenance, as well as cardiovascular and central nervous system functions. It is also one of the main drivers of tumorigenesis in breast and uterine cancer and can be targeted by several types of hormonal therapies. ERα is expressed in a subset of luminal cells corresponding to less than 10% of normal mammary epithelial cells and in over 70% of breast tumors (ER+ tumors), but the basis for its selective expression in normal or cancer tissues remains incompletely understood. The mapping of alternative promoters and regulatory elements has delineated the complex genomic structure of the *ESR1* gene and shed light on the mechanistic basis for the tissue-specific regulation of *ESR1* expression. However, much remains to be uncovered to better understand how *ESR1* expression is regulated in breast cancer. This review recapitulates the current body of knowledge on the structure of the *ESR1* gene and the complex mechanisms controlling its expression in breast tumors. In particular, we discuss the impact of genetic alterations, chromatin modifications, and enhanced expression of other luminal transcription regulators on *ESR1* expression in tumor cells.

## 1. Introduction

Breast cancer is the most frequent malignancy in women worldwide, with outcomes strongly affected by the stage of the disease [1]. It is characterized by the unregulated proliferation of epithelial cells normally located in the mammary ducts or alveoli. However, immunohistochemical (IHC) analysis reveals that breast cancer is a heterogeneous disease as evidenced by the differential detection of two main targetable tumorigenic drivers: estrogen receptor alpha (ERα), a nuclear receptor, and ERBB2/HER2, a membrane receptor belonging to the ERBB1-4 growth factor receptor family whose gene is frequently amplified in breast cancer. Use of positivity thresholds for detection of each marker by IHC (and for detection of ERBB2/HER2 amplification by fluorescence in situ hybridization) identifies four tumor subtypes, ER+HER2−, ER+HER2+, ER−HER2+ and ER−HER2− [2]. The expression of a third marker, the progesterone receptor (PR, NR3C3), an ERα target gene, reflects active estrogenic signaling and is a marker of improved prognosis compared to PR-negative tumors [3]. ER+ and/or PR+ tumors represent more than two-thirds of breast cancer cases and are currently treated with hormonal therapies that most commonly include aromatase inhibitors, which suppress endogenous estrogen production, or antiestrogens, which compete with estrogens for binding to estrogen receptors and inactivate them by inducing alternative conformations of their ligand-binding domains [4,5]. On the other hand, primary tumors with undetectable ER expression levels and activity are intrinsically resistant to hormonal therapies. These include ER-HER2+ tumors, and also tumors negative for ER, PR and HER2, called Triple Negative Breast Cancer (TNBC). It is estimated that approximately 30–50% of patients relapse after one or several lines of endocrine treatment. Expression of ERα is maintained in most relapsed tumors, which can remain sensitive to different hormonal therapy agents. However, about 15–40% of relapsed tumors lose expression of ERα and are therefore insensitive to additional hormonal treatment [6,7,8]. This review will discuss specifically the mechanism controlling ERα expression levels in normal and breast cancer cells. Other mechanisms of resistance to hormonal therapies, which include activation of other proliferative pathways and modulation of ERα or coregulator activity via genetic or epigenetic alterations, have been reviewed elsewhere (e.g., [7,9,10,11]).

**Figure 1:**
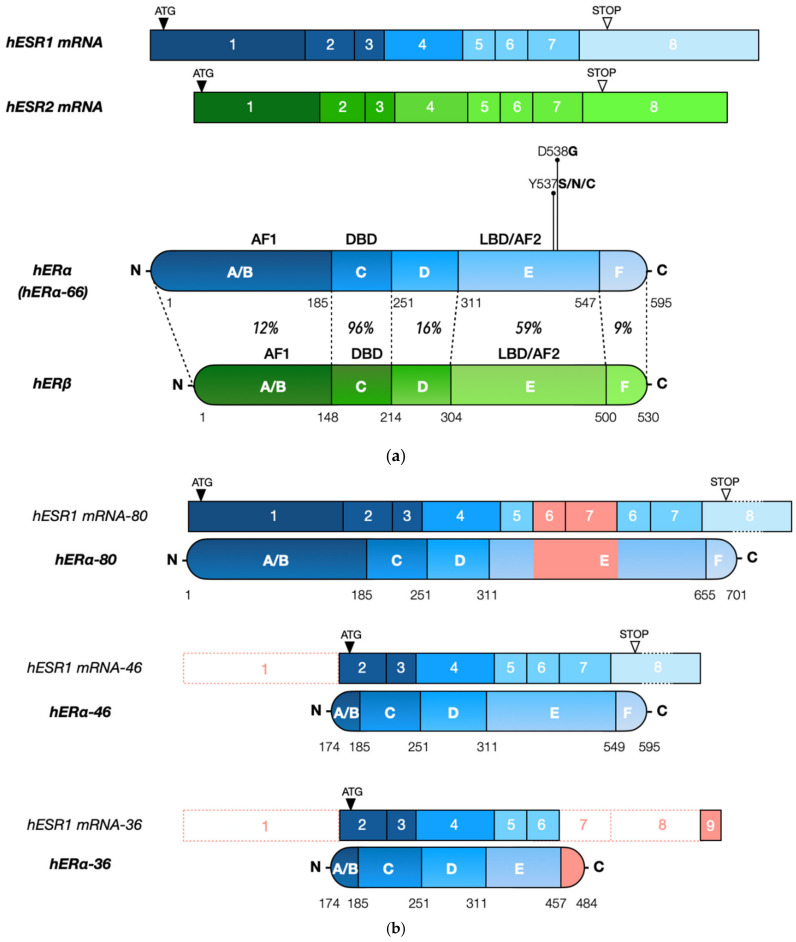
(**a**). ERα and ERβ have a conserved gene and protein organization. Amino acid identity between human ERα and ERβ is indicated for each domain [12]. The most frequent ERα mutations found in relapsed ER-positive tumors are indicated. (**b**). The main ERα protein isoforms and their corresponding mRNA structures are shown.

ERα and its paralog estrogen receptor beta (ERβ), encoded by the estrogen receptor 1 (*ESR1*) and the estrogen receptor 2 (*ESR2*) genes, respectively [12] (Figure 1a), mediate the systemic physiological effects of circulating estrogens [13]. ERα can be found in three major protein isoforms differing in molecular weight, ERα-66, ERα-46 and ERα-36 (Figure 1b), while ERβ has five isoforms, ERβ1-5. These receptors are part of the broader nuclear receptor family, composed of transcription factors whose activity can be regulated by small ligands and/or post-translational modifications [14]. Human ERs have only 44% overall identity in their primary structures but contain a well conserved DNA binding domain (96% identity between human ERα/β, Figure 1a) [15] composed of two zinc fingers folding into a single structural entity that directs binding of ER dimers to palindromic estrogen response elements (EREs) [16,17,18]. ER binding sites can be found at promoters of estrogen target genes but also at enhancers situated in intergenic or intronic sequences and having long-range impacts on gene transcription [19,20,21,22,23]. However, only a minority of predicted binding sites are used under any given experimental conditions [24], occupancy being controlled by chromatin accessibility [25] and/or cooperativity with other transcription factors, some of them acting as pioneer factors to enable access of ERs to cognate binding sites [26]. In addition to direct binding to EREs, ERs can also be recruited onto DNA by tethering via interactions with other transcription factors. Ligand addition leads to increased association of ERα with DNA and assembly of clusters of ERα-bound enhancers, or super-enhancers, organized around “mother enhancers” that are enriched in strong EREs and are detected in estrogen-deprived cells. By contrast, “daughter enhancers” appearing near mother enhancers upon ligand stimulation are enriched in sites for cooperating factors, suggestive of protein–protein interactions driven by the increased local concentrations of ERs on DNA [27]. Some enhancers are “hotspots” of colocalization of many transcription factors, which may be assembled in a “MegaTrans” complex marking the most active enhancers [28,29,30]. In particular, interaction with CTCF and components of the cohesin complex, which play roles in chromatin loop formation [31], has been described as a hall-mark of “hub” enhancers driving local chromatin conformation within topologically associated domains (TADs) [32].

The ligand binding domains of ERs are also conserved, although to a lower degree (59% identity between human ERα/β) [15], which has made it possible to design receptor-specific synthetic ligands [33]. Both receptors are nevertheless activated by circulating estrogens, which trigger conformational changes in the ligand binding domain inducing receptor binding to DNA and recruitment of a variety of transcriptional coactivators or co-repressors [12,15,34,35,36,37,38]. The two receptors diverge more in other domains (Figure 1a), and as a result differ in their functional properties [39] and in their regulation by post-translational modifications (please refer to Phosphosite.org for a summary of ERα/β post-translational modifications and to [11,40,41] for reviews). Modifications can affect diverse ER functional properties, including subcellular localization, DNA binding, interaction with other transcriptional regulators or chromatin components, and protein stability. Post-translational modifications include phosphorylation by diverse kinases at multiple sites in a manner regulated by ligand binding. In addition, ERs can be acetylated, methylated and SUMOylated in a ligand-modulated manner. In addition, ubiquitination can be induced by agonist binding but also by some antiestrogens, such as fulvestrant, the prototype of Selective Estrogen Receptor Downregulators (SERDs), resulting in enhanced degradation of ERα by the proteasome machinery [42]. Finally, palmitoylation regulates localization of a pool of ERα at the plasma membrane, where it initiates non-genomic estrogenic signaling that can cross-talk with the nuclear pool of ERα [43]; mouse models with a mutation abolishing palmitoylation support a tissue-specific contribution of membrane signaling in estrogen physiology, while lack of nuclear ERα abrogates most responses [34,39].

ERα is the main tumorigenesis driver in breast cancer, the roles of the ERβ isoforms being less clear, although it was shown that they can repress the activity of ERα [44]. The human *ESR1* gene, located at the q25.1-q25.2 locus of chromosome 6, has a complex structure. A cDNA was first cloned from MCF-7 cells in 1985 and was found to result from the transcription of eight exons, which together with introns span 296 kb [45,46,47,48]. However, characterization of transcripts in different estrogen-responsive tissues has revealed that the overall gene unit spreads over ~447 kb, comprising several alternative promoters and non-coding exons. Various transcriptional regulators bound to its distinct promoters or to enhancers acting at a large distance via the formation of chromatin loops control *ESR1* gene expression during the development, normal physiology and tumorigenesis of the mammary tissue and in other estrogen-responsive tissues [49]. In this review, we will focus on the mechanisms known to control *ESR1* expression, with a focus on positive regulation by transcription factors expressed in ER+ breast cancer, also called luminal breast cancer.

## 2. Expression of *ESR1* Is Transcriptionally Regulated in Normal Mammary Tissue and in Breast Tumorigenesis

### 2.1. Expression in Normal Tissue

The mammary gland, an organ uniquely found in mammals, is composed of an epithelial and a stromal compartment [50]. The first stage of mammary tissue development occurs during mammalian embryogenesis and leads to the formation of a rudimentary ductal tree connected to the nipple [50,51]. During puberty, the mammary tissue will further develop to form an expanded tree of epithelial ducts ending in terminal end buds, embedded in the mammary fat pad. Throughout pregnancy, the ductal tree further expands and lactogenic differentiation of luminal cells in alveoli enables milk production for lactation [51].

In normal human breast tissue, *ESR1* expression is restricted to the epithelial compartment, where ERα is detected in only about 7–10% of cells [52,53]. ERα is absent in differentiated myoepithelial cells and present only in a fraction of luminal cells in the human mammary gland where its expression levels are variable throughout life, particularly following puberty, during menstrual cycles and throughout pregnancy and lactation [54,55]. During mouse mammary gland development, ductal growth is initiated at terminal end buds (TEBs); ERα is not expressed in cap cells, located around the leading tip of the TEB, but is detected in a fraction of the underlying body cells, which also express luminal markers keratin 8/18 [51,56]. In mice, expression of the *Esr1* gene is necessary for the development and differentiation of the mammary gland, which is under control by estrogens and progesterone [57,58,59]. Indeed, suppression of *Esr1* expression disrupts the morphogenesis of the mammary gland and the function of the entire reproductive system [59,60].

The synthesis of sex hormones in the ovaries is regulated throughout the hormonal cycle after puberty and before menopause. While estrogens are produced during the entire hormonal cycle with a peak between the follicular phase and ovulation, progesterone is produced mostly during the second half (luteal phase). Increased expression of the *ESR1* gene in epithelial cells during the follicular phase correlates with subsequent increased mitotic activity of epithelial cells in breast lobules [61,62,63]. However, ERα expression in individual normal epithelial cells does not correlate with proliferation (KI67 positivity, tritiated thymidine incorporation) [64], and it has been shown that paracrine signaling via ERα stimulates mammary epithelial cell proliferation during mammary morphogenesis [65]. However, studies in rodents and rhesus monkey have indicated that ERα protein expression is down-regulated by estrogens, which may explain the apparent lack of association of ERα-expressing cells with proliferative markers [66,67]. In breast cancer cell lines, ERα drives cell proliferation by activating expression of proliferative genes, including *CCND1*, *MYC*, *E2F*s and *FOXM1* [68,69,70,71]. In ER+ MCF-7 breast cancer cells, ERα protein levels are highest in the S and G2/M phases of the cell cycle [72]. The proliferative action of estrogens in tumors as well as cell lines is confirmed by the observation that hormonal therapies suppress proliferation of breast tumor cells [73].

### 2.2. Expression in Tumoral Tissue

In the clinic, breast tumors have been deemed ERα-positive (ER+ tumors) if more than 1% of epithelial cells demonstrate nuclear staining in immunohistochemistry with anti-hERα antibodies, qualifying patients for hormonal therapies, although rates of response are much lower for tumors with low expression of ERα [74]. While there is a range of ERα expression patterns between tumors, expression levels of the ERα protein are often higher and more homogeneous than in normal tissue, especially in the LumA subgroup. Nevertheless, some tumors exhibit heterogeneous expression, with variable proportions of cells expressing ERα while staining is faint or absent in others.

Elevated *ESR1* expression has been observed in atypical ductal hyperplasia and in situ carcinoma, suggesting that deregulation of the *ESR1* expression level may be implicated in early pathogenic changes during breast tumorigenesis [62,75]. Whether *ESR1* overexpression is sufficient to drive tumorigenesis in the human mammary gland is unclear. Interestingly, transgenic expression of wild type (wt) murine ERα in mice using an MMTV-tTA controlled expression system led to ductal hyperplasia and ductal carcinoma in situ by four months of age and to a low incidence of invasive carcinoma by 12 months [76,77]. However, it remains unclear whether deregulated *ESR1* expression is sufficient to drive tumorigenesis in the human mammary gland.

**Figure 2:**
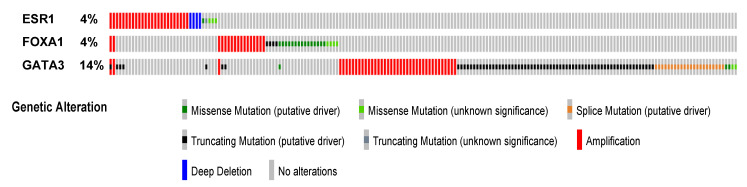
Genetic alterations in *ESR1*, *GATA3* and *FOXA1* in the TCGA Firehose Legacy breast invasive carcinoma dataset. Amplification, deep deletion or mutations in genes for luminal transcription factors ERα, FOXA1 and GATA3 are shown. The figure was downloaded from cbioportal.org by selecting the TCGA Firehose Legacy dataset, querying the *ESR1*, *FOXA1* and *GATA3* genes and using the OncoPrint tool.

**Figure 3:**
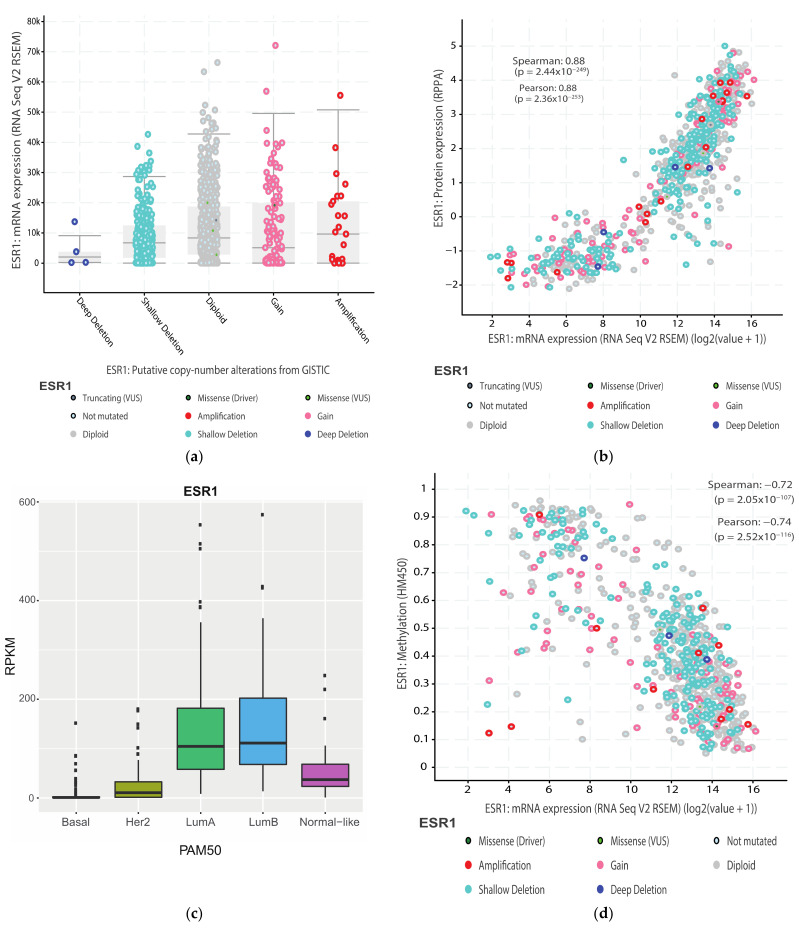
Expression of the *ESR1* gene is epigenetically controlled (**a**). Impact of CNVs on *ESR1* expression in the TCGA Firehose Legacy breast invasive carcinoma dataset. (**b**). Correlation between *ESR1* mRNA and protein expression in the same dataset. (**c**). Differential expression of *ESR1* within PAM50 breast tumor types (**d**). Correlation between *ESR1* gene expression and DNA methylation in the first intron in the TCGA Firehose Legacy breast invasive carcinoma dataset. Panels (**a**,**b**,**d**) were downloaded from cbioportal.org by querying the *ESR1* gene and using the Plots tool. Panel c was produced using the PAM50 classifier on the TCGA breast invasive carcinoma dataset in [99].

The *ESR1* gene is affected by copy number variations, with amplification events initially reported at a high frequency (20%), correlating with higher ERα levels [78]. On the other hand, Brown et al. found amplification in only 1% of tumors [79]. Amplification is predicted by GISTIC in less than 4% of tumors in the TCGA Firehose Legacy dataset (Figure 2) and at similar low frequencies in other large breast tumor collections such as METABRIC. However, amplification does not correlate strongly with increased mRNA (Figure 3a) or protein expression. ERα is not frequently mutated in primary cancer, while recurrent mutations occur in a fraction of tumors progressing to resistance after hormonal treatment [80]. Several of these mutations (e.g., hot spot mutations Y537S/N/C and D538G, Figure 1a), lead to constitutive ERα activity, which generates resistance to aromatase inhibitors. ERα mutations can also differentially impact the potency and/or efficacy of clinically-relevant antiestrogens [80,81,82,83,84].

In primary tumors, *ESR1* mRNA expression correlates very well with protein expression, suggesting that regulation of *ESR1* expression takes place mainly at the RNA level (transcription and/or mRNA stability) or that a balance between protein and RNA levels is maintained via tight feedback regulation (Figure 3b). The *ESR1* mRNA is differentially expressed in breast cancer subtypes (Figure 3c). In the transcriptome-based PAM50 classification, *ESR1* expression is highest in the luminal A and luminal B tumors, the latter being associated with higher grade and worse prognosis [85]. On the other hand, the HER2+ tumors express the *ESR1* mRNA at variable and overall weaker levels while the basal-like subtype has very weak to undetectable levels of *ESR1* mRNA and of the ERα protein (Figure 3c). The existence of discrete “intrinsic” subtypes with differential expression of the *ESR1* gene likely reflects the phenotype of the cell of origin giving rise to these tumors (stem cell, bi-valent or univalent progenitor) and the inhibitory impact of genetic/epigenetic aberrations in breast cancer cells on normal epithelial cell differentiation.

*ESR1* expression is rapidly lost in primary cultures of normal epithelial cells, either through epigenetic silencing or lack of proliferation/death of ER-positive cells. In addition, only a handful of ERα-expressing cell lines have been derived from ER+ tumors. ER+ tumors are also more difficult to engraft in Pdx models. Thus, our knowledge of ER signaling in tumors obtained from existing models may be biased toward more aggressive tumors. Nevertheless, ER+ cell lines such as MCF-7, T-47D and ZR-75-1 cells have proven very useful to dissect pathways regulating ERα expression as well as its role in the control of BC cell proliferation [86].

**Figure 4:**
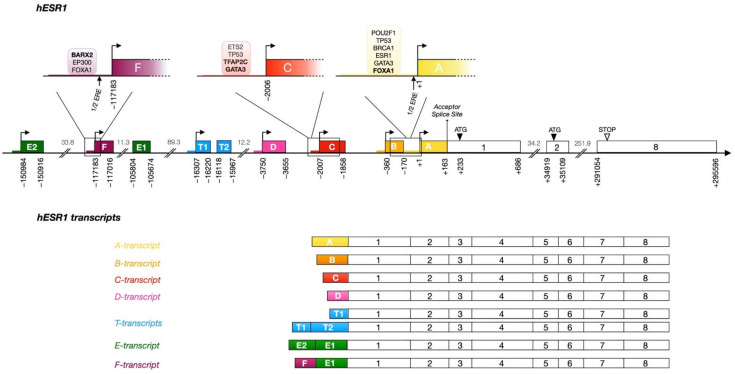
Schematic structural organization of human *ESR1* promoters and alternative transcripts. The structure of the human *ESR1* gene is presented along the main axis, boxes corresponding to exons. Alternative upstream exons are colored and labeled with letters according to the nomenclature proposed by Kos et al. [49]. White boxes correspond to exons downstream of the common acceptor splice site and are numbered according to the same nomenclature. Alternative promoters are represented by a colored thick line matching the color of the corresponding 5′ exon. The arrows define transcriptional initiation sites. The numbers under the main axis are exon start/end distances from the transcription start site originally defined as +1 (transcript A), calculated based on mapped transcripts in the hg19 genome version. The numbers between the exons represent the size of the introns in kilobase pairs. The common acceptor splice site is represented by a vertical bar before exon 1 and the ATG start codon is indicated by a black arrow. The upper part of the diagram details the promoters of exons A, C and F. Reported half-estrogen response elements (1/2 EREs) in these promoters are indicated by arrows. Transcription factors bound in these regions based on published ChIP-Seq data are shown in bubbles and factors directly binding DNA (motif predicted by HOMER) are in bold. The different transcripts produced after transcription and splicing from each of the seven regulated upstream exons to exon 1 are also presented in the lower part of the diagram.

Methylation studies of the *ESR1* gene have demonstrated that a CpG region located from the acceptor splice site in the first coding exon to the end of this exon (exon 1, Figure 4) is unmethylated in ER+/PR+ tumors and in ER+ cell lines MCF-7, T-47D and ZR-75-1, while it is partially methylated in ER− MDA-MB-468 cells and completely methylated in mesenchymal MDA-MB-231 cells [87]. Surprisingly, ER+/PR- tumors also presented some methylated CpG sites [87]. These results may be explained by the inclusion in the ER+ group of tumors with low ERα levels, resulting in undetectable expression of PR, whose gene is under estrogenic regulation. In addition, *ESR1* expression is strongly negatively correlated with DNA methylation downstream of the major transcription start site in the TCGA breast tumor dataset (Figure 3d). Treatment of triple negative cell lines with DNA methyltransferase inhibitors such as 5′aza-deoxycytidine and/or histone deacetylase (HDAC) inhibitors was reported to induce expression of the *ESR1* gene, leading to sensitivity to tamoxifen [88,89,90,91]. However, other studies have failed to reproduce these observations [92]. On the other hand, treatment of ERα-expressing breast and uterine cancer cells with HDAC inhibitors leads to suppression of *ESR1* expression [93,94,95,96]. Recently, treatment of triple negative breast tumor cells MDA-MB-231 LM2 with EZH2 inhibitors has been shown to induce *GATA3* expression and, more modestly, *ESR1* expression, and to sensitize these cells as well as another TNBC cell line (MDA-MB-468) to treatment with the antiestrogen fulvestrant [97].

These observations suggest that epigenetic control through DNA methylation and histone acetylation/methylation plays a role in *ESR1* expression in breast cancer cells. In addition to this, mutations activating or inactivating one of the multiple regulatory elements in the *ESR1* gene [98], from changes in expression/activity of upstream regulators (see below), or a combination of both, may also contribute to the regulation of *ESR1* expression. Altogether, the existence of different mechanisms of *ESR1* gene expression activation likely contributes to the heterogeneity of *ESR1* expression levels and patterns between and within tumors.

## 3. *ESR1* Gene Organization and Regulatory Sequences

### 3.1. ESR1 Gene Structure and Alternative Transcripts

The analysis of different cDNA clones for the *ESR1* gene has led to the characterization of seven promoters controlling nine upstream exons (A, B, C, D, T1/T2, F, E2/E1, Figure 4) [49,100,101]. The splicing of exons E1, T1, T2, D, C, and B to a common acceptor splice site located at position +163 in the first exon transcribed from promoter A yields 7 *ESR1* transcript isoforms that can all be translated into the same 66 kDa protein [49,100,101]. The seven alternative promoters are regulated in a tissue-specific manner and generate transcripts with unique 5′ untranslated regions (5′ UTRs).

Nevertheless, several isoforms of the ERα protein can result from alternative splicing. A truncated form of ERα with an alternative, shorter N-terminal end, ERα-46 (Figure 1b), has been identified in breast cancer cells. It results from transcription initiated at the E2 or F exons, spliced to exon E1 and then directly to exon 2, deleting exon 1 [102,103]. Expression of this isoform is regulated by specific transcription factors [104,105]. The homeobox transcription factor BARX2 has been shown to bind to the *ESR1* gene at the level of the E1 and F promoters and to upregulate transcription of this isoform [104]. The high mobility group A protein 1a (HMGA1a) is involved in the alternative splicing mechanism of this transcript by the recruitment of the RNA-protein complex U1 snRNP at the acceptor splice site [105]. ERα-46 translation is intiated at an ATG codon in a favorable Kozak consensus sequence in exon 2 and lacks most of the AF1 transcriptional activation domain, acting in a dominant negative manner to suppress genomic estrogen signaling and estrogen-dependent proliferation [106]. Furthermore, a shorter 36 kDa isoform has been characterized [107]. This protein, encoded by exons 2 to 6 and an alternative exon 9, located 64 kbp downstream of the *ESR1* gene, is truncated both in the AF1 (lack of exon 1) and AF2 (lack of exons 7–8) transcriptional activation domains but preserves the DNA binding domain, as well as a truncated ligand binding domain with a different 27 aa C-terminus. The function of these truncated isoforms is not yet fully elucidated, although different lines of research suggest both short isoforms are localized preferentially at the plasma membrane [108]. This may result from the absence of exon 1, placing three potential myristoylation sites at residues 25–30 (GVWSCE), 76–81 (GMMKGG), and 171–176 (ELLTNL) in both shorter isoforms, and therefore much closer to the N-terminus of the protein than in the full length ER [107]. Moreover, the expression level of ERα-36 has been shown to correlate with a worse prognosis, the proposed mechanisms being, in spite of the LBD truncation, estrogen-dependent stimulation of mitogen signaling activity, activation by antiestrogens such as tamoxifen, and increased stemness and metastasis of breast cancer through upregulation of the aldehyde dehydrogenase 1A1 (*ALDH1A1*) gene [108,109].

Rearranged forms of the *ESR1* gene have also been described. A duplication of exons 6 and 7, encoding part of the E domain, has been reported, leading to an ERα isoform of 80 kDa [110]. This longer isoform was found in a subclone of the MCF-7 cell line, MCF-7:2A, which contains four to five copies of the *ESR1* gene and is estrogen independent. Moreover, 88 *ESR1* gene fusions with a break point in or near intron 6, resulting in truncation of the LBD, have been characterized in metastatic ER-positive disease with a frequency estimated at more than 1% [111]. Contrary to hERα-36, which lacks exons 7 and 8 but was found to be activated by estrogens [108], these fusions are reportedly devoid of estrogen-dependent activity but have different levels of constitutive activity dependent on their fusion partners.

### 3.2. ESR1 Alternative Promoters Have Tissue-Specific Activity

Alternative transcripts of the *ESR1* gene have been detected in both normal and cancerous tissues. In human cell models of ER+ breast tumors, *ESR1* expression is driven predominantly by the proximal A promoter and the C promoter located 1.9 kb upstream [112]. Experiments from Grandien et al. demonstrated that both the A and C mRNA isoforms were also expressed in endometrial tissue and uterine adenocarcinoma cell lines. Interestingly, the mRNA levels of the A and C transcripts are widely different in breast cancer cells, with a ratio of approximately 20:1 [112]. The A transcript is more highly expressed in breast tumors than in the normal mammary gland, but the opposite is observed for the C mRNA isoform [112]. All transcripts except the T-transcript have been cloned from the MCF-7 cell line and have been shown to be expressed in the mammary gland, in endometrium and/or in liver [112].

**Promoter A:** Despite a degenerate TATA box (TACTTAAAG), the A transcript is the most abundant, whether in healthy mammary tissue or in luminal tumors [113,114]. DNA methylation analyses performed on a large panel of breast cancer cell lines revealed that none of the 22 CpG sites identified in promoter A were methylated [115]. However, data from ENCODE indicates that these sites are highly methylated in the HeLa cell line, consistent with the lack of expression of *ESR1* in these cells.

ENCODE ChIP-Seq data reveals binding of several transcription factors (TFs) within 500 bp of the transcriptional start site (TSS) of promoter A. FOXA1, GATA3 and ERα interact with the promoter in the T-47D BC cell line and CTCF, MYC and POLR2A interact in the MCF-7 cell line. A predicted binding site for FOXA1 coincides with a FOXA1 ChIP peak 400 bp upstream of the promoter A TSS. The existence of a half ERE in promoter A (arrow, Figure 4) could possibly contribute to the recruitment of ERα [114,116]. ChIP-Seq assays targeting the breast cancer type 1 susceptibility protein (BRCA1) have shown that this protein binds the A promoter indirectly via Oct-1, encoded by the *POU2F1* gene [117]. Oct-1 binds a region of promoter A between -385 and -169 bp and recruits BRCA1 as a coactivator, resulting in positive transcriptional regulation of *ESR1* [117]. In addition, functional genomic studies have shown the presence of the p53 protein in the region between −128 to −40 bp of promoter A, accompanied by other factors such as Sp-1, c-Jun and the coactivator CBP, suggesting regulation of *ESR1* by p53 via this promoter [118]. This is consistent with the higher rate of p53 mutations in ER-negative vs. ER-positive and in luminal B vs. luminal A tumors, although the extent to which p53 controls *ESR1* expression remains unclear. The recruitment of Sp-1 within the −245 to −192 bp region was found to be essential for *ESR1* expression and to mediate positive regulation of promoter A by exogenous Sp-1 expression in Drosophila Schneider SL2 cells [119].

The presence of the repressive histone methylation mark H3K9me3 has been reported at promoter A in basal breast cancer cells. ChIP-qPCR on FOXC1 wt and knock-out BT549 basal-like breast cancer cells showed a correlation between trimethylation of H3K9, FOXC1 expression and loss of RNA PolII recruitment at the *ESR1* regulatory sequence. This negative regulation of *ESR1* gene expression reinforces the hypothesis that *ESR1* heterogeneity between different breast cancer types is due to the establishment of distinct epigenetic marks [120].

**Promoter B:** As there are only about 360 bp between the B and A transcript start sites, several of the TF binding sites upstream of promoter A overlap with promoter/exon B. The B transcript has been detected in breast tissue at very low mRNA levels compared to the A transcript. The B promoter and exon B include 18 CpG sites that are unmethylated both in normal MCF-7 cells or in antiestrogen and anti-aromatase inhibitors resistant MCF-7 cells, which express lower levels of ERα [115].

**Promoter C:** Exon C presents two main transcription start sites located at −1974 and −2007 bp, generating C-transcripts with different 5′-ends [113]. C transcripts are ten times less abundant than the A-transcript in MCF-7 cells and absent from ZR-75 cells. Promoter C is highly methylated in ZR-75-1 cells, correlating with the absence of the C-transcript in that cell line, while it is unmethylated to moderately methylated in ER-negative SK-BR-3 and MDA-MB-231 cells [115].

ENCODE ChIP-Seq data and literature reports suggest binding of multiple TFs to this promoter in MCF-7 cells. The activity of the promoter C in luminal cell lines was observed to depend on the binding of estrogen receptor associated factor 1 (ERF-1), corresponding to the transcription factor AP-2 gamma (TFAP2C) [121,122]. This factor binds promoter C at −1877 bp in a complex with Jun/Fos and plays an important role in the expression of the *ESR1* gene [123,124,125]. Sodium bisulfite genomic sequencing revealed that promoter C comprises nine CpG sites [115,121]. The ninth methylation site is located in a TFAP2C binding motif [115,121]. *TFAP2C* is expressed in both the luminal and myoepithelial cells in the adult mammary gland and is expressed in all BC subtypes [126]. MMTV-Cre induced knockout in mouse models leads to delayed migration through the mammary fat pad without deleterious impact on mammary gland function, to an increase in the CD24midCD49fhi population, an increase in basal CK5 staining and a decrease in the normalized luminal:basal ratio. Down-regulation of *TFAP2C* in ER+ BC cells leads to epithelial to mesenchymal transition (EMT) characterized by increased *VIM* and *CD44* expression, with decreased *CDH1* and *CD24* expression and decreased luminal gene (*ESR1*, *FOXA1*, *GATA3*) expression [127]. TFAP2C also regulates *FOXM1* expression and its down-regulation suppresses estrogen-dependent proliferation [128]. The related TFAP2A on the other hand specifically induces CDKN1A and is inactive on luminal genes [129].

Specific methylation of the fourth CpG site in promoter C was responsible for a decreased expression of *ESR1* and resistance to endocrine therapy; the absence of methylation at this specific CpG site allows the recruitment of the methylation-sensitive transcription factor Ets-2, which leads to *ESR1* expression in normal MCF-7 cells [115,121]. In the MCF-7 cell line, GATA3 and the negative regulator FOXC1 were reported to compete for binding to DNA at overlapping recognition motifs [120]. Induced expression of FOXC1 in MCF-7 cells leads to a loss of GATA3 recruitment to promoter C, indicating that FOXC1 can abolish the positive regulation of that promoter [120]. More distally, CTCF also binds promoter C in the MCF-7 cell line. Finally, p53 is recruited at the C promoter in ChIP-qPCR assays in the DNA region between −2094 to −1941 bp from the main TSS, which contains seven of the nine methylation sites described previously [118,124,130].

**Promoter D:** Promoter D was shown to be fully methylated in the MCF-7 cell line. DNA methylation studies identified seven CpG sites methylated in that promoter, but the expression level of the D-transcript did not correlate with the methylation status [115]. Moreover, an AP-1 binding site has been described as an enhancer active in MCF-7 cells and inactive in ER-negative MDA-231 cells [125].

**Promoter F:** The F-transcript, produced from the splicing of exons F and E1, is the predominant variant expressed in primary osteoblasts but is also highly expressed in breast cancer tissue [114,131]. As mentioned above, promoter F was found to be regulated by BARX2 in breast normal and tumoral tissue [104]. FOXA1, GATA3 and p300 also interact with this promoter in the MCF-7 and T-47D cell lines, correlating with the levels of F-transcripts in ER-positive breast cancer cells [132]. The existence of a half ERE in promoter F (arrow, Figure 4) was suggested to contribute to the recruitment of ERα [114,116]. E2F1 is also associated with promoter F in ENCODE MCF-7 ChIP-Seq data. DNA methylation analysis in this promoter has shown the presence of 10 CpGs, unmethylated in MCF-7 but all methylated in the MDA-MB-231 cell line [132].

**Promoter E:** The E-transcript is produced by the transcription of exons E2 (upstream of promoter F) and E1 (downstream of promoter F) and is expressed mainly in the liver and at very low levels in breast cancer cells [114].

**Promoter T:** This promoter is the only one not to be active in mammary glands or in breast tumors. The T-transcript is predominantly expressed in testis and epididymal tissues. The T1 and T2 exons are separated by a 101 bp intron and are located about 16 kb upstream of the first exon [49,133]. Transcription from the T promoter can form either a longer variant from the splicing of exon T1 to exon T2 and finally to exon 1, or a shorter variant from the splicing of the T1 exon directly to exon 1.

### 3.3. Upstream Enhancers

**Figure 5:**
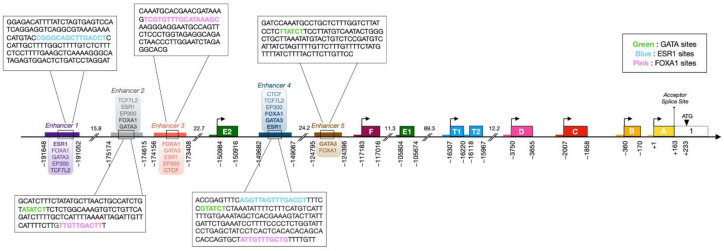
Enhancer regions of the human *ESR1* gene bound by luminal TFs. Colored rectangles highlight the luminal enhancers defined by ChIP-Seq assays with luminal factors ERα, FOXA1 and GATA3 and coactivator p300 [19,135,136,137]. Alternative exons spliced with coding exon 1 are shown as in Figure 4. The promoters are represented by a colored thick line upstream of the corresponding alternative first exons. Broken arrows define the different transcription initiation sites. Distances between the beginning or end of each defined feature and the transcription start site in promoter A are indicated. The distance in kilobase pairs between two highlighted elements is shown under the DNA axis. The common acceptor splice site is represented by a black line and the ATG start codon is indicated by a black triangle. Transcription factors bound to each enhancer based on ChIP-Seq data are shown in bubbles above or below each element, and factors for which a recognition motif is predicted by HOMER or mapped in a referenced study are in bold. DNA sequences from the enhancers containing one or more binding motifs for GATA3 (Green), FOXA1 (Pink) and/or ERα (Blue) are shown in magnification boxes (motifs for each TF are in bold).

Distal enhancers can regulate transcription at up to several hundred kilobases away from target genes via chromatin looping [134]. ChIP experiments have revealed binding of luminal TFs FOXA1, GATA3, and ERα itself to far upstream regulatory sequences in the *ESR1* gene [19,135,136,137]. All regions interacting with these luminal TFs are associated with enhancer chromatin marks H3K4Me1 and H3K27Ac in ENCODE datasets generated in MCF-7 cells, supporting the existence of at least five “luminal” enhancers located at distances greater than 120 kb upstream of exon A (Figure 5; enhancers 1–3 are upstream of exon E2, enhancers 4 and 5 are found between exons E2 and F). FOXA1 and GATA3 bind to enhancers 1–5 and ERα to enhancers 1–4. Thus, ERα, GATA-3 and FOXA1 appear to share *cis* regulatory sites in the *ESR1* gene as well as upstream of many estrogen target genes [19,135,136,137]. Enhancers 1 and 4 contain motifs related to the consensus ERE sequence 5′-(A/G)GGTCANNNTGACC(T/C)-3′ [138,139,140]. Predicted FOXA1 motifs related to the consensus 5′-A(A/T)TRTT(G/T)RYTY-3′ [141] are found in enhancers 2, 3 and 4. Sequences matching the consensus GATA binding motif 5′-(A/T)GATA(A/G)-3′ [142,143,144] are predicted by HOMER in enhancers 4 and 5. A reported GATA3 site [98] not predicted by HOMER is located in enhancer 2. Thus, one or more predicted binding motifs for ERα, FOXA1 and GATA3 are found in each of the five enhancers. While ERα, FOXA1 and GATA3 are detected at regions that do not contain mapped binding motifs, lower affinity sequences may be accessed through DNA binding cooperativity and/or pioneer activity. Alternatively, these TFs may be tethered to other TFs directly recruited to DNA. Other transcription factors are also present at these sites as annotated in ENCODE ChIP-Seq datasets in MCF-7 cells, suggesting that they correspond to active enhancers [30]. The coactivator p300 (EP300), which acts as a general transcriptional coregulator with histone acetyltransferase activity [145], and is present at 51% of the ERα binding sites after E2 treatment [146], is strongly recruited to enhancers 1–4, as well as RNApolII. While these enhancers are not clustered in super-enhancers, we note that enhancers 3 and 4 are associated with CTCF and components of the cohesin complex, corresponding to the profile of “hub” enhancers [32], which play major roles in local chromatin organization. Finally, chromatin loops were observed to form between enhancers 1 and 2/3, 1 and 4, 2–3 and 4, and 1 and promoter F by ChIA-PET with ERα in MCF-7 cells [147]. Looping with other regions not strongly associated with luminal factors in MCF-7 cells was also observed, suggesting that other regulatory sequences play roles in *ESR1* transcription, in keeping also with the existence of several other regions associated with H3K4Me1/H3K27Ac and DNAse hypersensitivity.

Breast cancer risk-associated single nucleotide variants (SNVs) and somatic mutations have been mapped to luminal enhancers and other DNAse hypersensitive sites within 1 Mb of the *ESR1* start site [98]. The rs9383590 SNV affects the reported GATA3 binding site in enhancer 2 and is predicted to increase activity of the enhancer. This suggests that this GATA3 element acts as a negative rather than positive regulator of *ESR1* expression. Of interest, this motif is located within a TCF7L2 ChIP binding region (ENCODE). The transcription factor TCF7L2 has binding patterns in MCF-7 cells that coincide with those of GATA3, resulting in repression of their common target genes [148] (see also below). In addition, mutation chr6:151979547:A>G in enhancer 4 affects a nucleotide within a predicted FOXA1 motif (5′ ATTGTTTGCTG 3′) [98]. While it is unclear whether this mutation affects FOXA1 binding, residues flanking the core FOX motif (5′-RTAAAYA-3′) can alter FOX factor binding specificity (see below). Point mutations that led to increased activity in reporter gene assays were also found in tumors between enhancers 2 and 3 (chr6:151954506 C>A), after enhancer 3 (chr6: 151955192A>G; chr6:151955219:G>T), downstream of the E2 promoter (chr6:152024472C>G) or upstream of promoter C (chr6: 152125116G>C); these mutations may suppress binding by transcriptional repressors or create new binding sites for transcriptional activators, suggesting that a variety of transcription factors bound to different regulatory regions regulated *ESR1* expression. Based on their data, Bailey et al. predicted that mutations leading to increased enhancer activity may explain the sustained expression of *ESR1* in some (about 7%) luminal breast tumors [98].

## 4. Luminal ESR1 Transcriptional Regulators

### 4.1. GATA3

The *GATA3* gene, located at 10p14 in the human genome, codes for a 48 kDa protein composed of two N-terminal transactivation domains, two zinc-finger DNA-binding motifs followed by two highly conserved and distinct basic regions (basic region 1 and 2) and a C-terminal region required for GATA3 transactivation, which contains a conserved YXKXHXXXRP motif [144,149,150]. The GATA proteins form a family of six zinc finger DNA binding proteins that recognize motifs related to the consensus sequence 5′-A/TGATAA/G-3′ [142,143,144]. GATA3 was shown to bind DNA with its C-terminal zinc-finger region and form homodimers, or heterodimers with other GATA members via its N-terminal zinc-finger region [144,151]. On certain specific DNA sequences, two close GATA sites can be bound simultaneously by the two zinc fingers of one GATA3 molecule with higher affinity [144]. Like ERα, GATA3 interacts with the acetyltransferase CREBBP/CBP, which acetylates it at K119, contributing to its capacity to downregulate mesenchymal transcription factors ZEB1/2 and Slug [152].

In breast tumors and cell lines, expression of *GATA3* is strongly associated with those of *ESR1* and *FOXA1* [153]. On the other hand, *GATA1, 2, 4* and *5* are only mildly or not correlated, while *GATA6*, the only *GATA* gene whose expression is biased toward basal-like tumors, is mildly anti-correlated with *ESR1* in the TCGA dataset [99]. GATA3 was identified as a regulator of mammary branching morphogenesis by genome-wide transcript analysis [154] and shown to maintain the differentiation of luminal cells in mouse models [155,156,157]. High levels of expression of *GATA3* in ER-positive cells are consistent with the reported positive cross-regulation feedback loop between the *GATA3* and *ESR1* genes [158], which also likely involves the FOXA1 luminal transcription factor. As mentioned above, GATA3 interacts with the *ESR1* promoters A and C but also with the five enhancers associated with FOXA1 in ChIP-Seq datasets [120,136] (Figure 5). Conversely, an enhancer recruiting ERα, FOXA1 and GATA3 itself, together with the coactivator p300, was described approximately 10 kb downstream of the *GATA3* gene (about 30 kb downstream of the TSS) [158]. Another region located 53.4 kb downstream of the gene (73.4 kb downstream of the TSS) is even more strongly associated with all factors in relevant ChIP-Seq/Exo datasets [136,137] and contains a predicted ERE and several FOX and GATA motifs, supporting the existence of regulatory loops between these factors.

Consistent with the role of GATA3 in the regulation *ESR1* expression, *GATA3* silencing by two different siRNAs was observed to reduce *ESR1* expression in T-47D and MCF-7 cells, to blunt induction of transcription of estrogen target genes, and to inhibit estrogen-induced proliferation [158]. On the other hand, expression of *GATA3* in MDA-MB-231 cells results in reprogramming of these cells from a mesenchymal to a luminal subtype, associated with a reduction of tumorigenesis and metastasis in implanted xenograft assays; GATA3 also induces a growth inhibitory response to TGFβ [159,160].

Moreover, GATA3 may act as a pioneer factor by binding target sites within nucleosomes and making neighboring cis-regulatory elements accessible for the recruitment of additional transcription factors [161,162,163]. During the mesenchymal-to-epithelial transition induced by GATA3, cellular reprogramming involves in part binding to closed chromatin regions, nucleosome eviction and chromatin remodeling in a transcriptional activation domain-dependent manner [162]. In ER-positive breast cancer cell lines, enrichment in GATA motifs was observed in ERα and FOXA1 ChIP-Seq/Exo peaks [136,137] and *GATA3* silencing decreased or even abolished the recruitment of ERα at distal regulatory regions normally co-occupied with GATA3 [161]. Recruitment of common cofactors may contribute to complex formation between GATA3 and other luminal TFs. GATA3 may also recruit other transcription factors by tethering. GATA3 expression was shown to be required for the high mobility group box-containing factor TCF7L2 to bind to about 50% of TCF7L2 sites in the ER-positive breast cancer cell line MCF-7, and these sites were enriched in GATA3 but not TCF7L2 motifs [148]. GATA3 and TCF7L2 bound to shared sites simultaneously and tagged TCF7L2 could co-immunoprecipitate with endogenous GATA3 in transfected MCF-7 cells. TCF7L2 functioned mainly as a transcriptional repressor at shared sites [148]. TCF7L2 is recruited at the level of enhancers 1, 2 and 4 of the *ESR1* gene; however, whether TCF7L2 represses *ESR1* expression needs to be confirmed.

*GATA3* is the third most mutated gene in luminal breast cancers with a prevalence of approximately 14% and 15% in luminal A and B tumors, respectively (cbioportal.org, TCGA Firehose Legacy dataset). *GATA3* is also amplified in breast tumors (Figure 2), mostly in ER-negative tumors where amplification correlates with decreased rather than increased *GATA3* expression, suggesting the existence of different driver genes in this 10p14 amplicon [164]. On the other hand, GATA3 is frequently affected by mutations in ER+ BC, with a bias for invasive ductal carcinoma, including splicing mutations (mostly X308_splice) and truncating frameshift mutations (in or after the second zinc finger) [165,166]. *GATA3* mutations can lead to active forms of the GATA3 protein that contribute to tumor growth in BC cell xenografts and promote precocious lobuloalveolar development in transgenic mice [167]. In addition, ER-positive breast tumors with *GATA3* mutations were reported to have a worse prognosis compared to wt tumors [168]. In the MCF7 cell line, GATA3 is affected by a heterozygous D336fs mutation in the second zinc finger that leads to a truncated protein with reduced affinity for DNA. Both wt and mt GATA3 have increased stability in MCF-7 vs. wt GATA3 in T-47D cells, leading to increased genomic occupancy by GATA3; genomic distribution was similar as in wt T47D but with gene-specific loss of binding, such as at the progesterone receptor gene [169]. Mutation R330fs was on the other hand reported to alter genomic distribution of ERα and FOXA1, leading to changes in gene regulation affecting genes involved in mammary gland development and epithelial cell biology [170]. In contrast, the X308_splice mutation, which generates a GATA3 protein lacking the second zinc finger but containing a novel 44 aa C-terminal end, downregulates ER genomic signaling and is associated with improved overall survival in ER+ patients [171].

### 4.2. FOXA1

The forkhead box (FOX) family of transcription factors comprises 50 human members that share a characteristic ‘winged-helix’ DBD composed of a helix-turn-helix motif with two C-terminal loops or “wings” [172] and regulate gene expression spatially and temporally during development [173]. These transcription factors can be divided into 19 subgroups (FOXA to FOXS) based on the sequence of their DBDs. Although all FOX proteins recognize core motifs related to the consensus 5′-RTAAAYA-3′, flanking sequences contribute to DNA binding specificity within the family [172,174]. Further, divergence in FOX factor primary sequence outside of the DBD results in a wide range of biological functions. In particular, FOXA1 plays an essential role in mammary ductal morphogenesis [175], and its expression is strongly correlated with that of ERα in breast luminal tumors [153]. FOXA1 has been identified as an upstream regulator of *ESR1* expression in mouse and human breast cancer cells [175,176] and as a repressor of basal-like genes [177]. Notably, FOXA1 is co-associated with GATA3 at several enhancers in the *ESR1* gene (Figure 5) and may thus cooperate with it for regulation of *ESR1* expression. In addition, FOXA1 can act as a pioneer transcription factor, directly binding and opening condensed chromatin [178,179]. FOXA1 can enable recruitment of ERα and the androgen receptor (AR) to their respective response elements in breast and prostate cancer, respectively [19,26,180,181,182]. Beyond facilitation of hormone receptor signaling, *FOXA1* overexpression has been shown to contribute to tamoxifen insensitivity of breast cancer cells in a mechanism that involves induction of interleukin-8 [183].

The *FOXA1* gene is infrequently amplified (Figure 2), and copy number gain does not associate with markedly higher expression levels, while shallow deletion and DNA methylation may account for low *FOXA1* expression (cbioportal.org). Mutations in FOXA1 are less frequent than those in GATA3 and are mostly missense mutations associated with high *FOXA1* mRNA levels. Contrary to GATA3 mutations, they are frequent in invasive lobular carcinoma [166]. These mutations affect mostly the forkhead domain, the most common in primary tumors being I176M/V, D226G/N and S250F (cbioportal.org, Breast invasive carcinoma, TCGA Firehose Legacy dataset). Mutations are enriched in metastatic tumors and correlate with resistance to aromatase inhibitors. Mutations in the Wing 2 region increase cooperativity with ER while mutation SY242CS, located in the third beta strand, leads to altered DNA binding patterns [184]. In addition, a G>A mutational hotspot was discovered in ER+ breast tumors at position -81 relative to the *FOXA1* TSS, resulting in increased binding of E2F1 and *FOXA1* overexpression [185]. Thus, enhanced expression or activity of FOXA1 could in turn result in increased ERα levels via direct transcriptional regulation of *ESR1* expression, in addition to the role of FOXA1 in cooperating with ERα for transcriptional regulation.

FOXA2 and FOXA3 are the closest homologs of FOXA1 in the FOX family. RNA levels of both factors are much lower than those of FOXA1 in breast cancer, and neither are positively correlated with *ESR1* expression. FOXO family members are also expressed in breast tumors, without a strong bias for luminal tumors. Activity of these factors is under post-translational regulation. For instance, FOXO protein expression is downregulated by the Akt pathway [186]. FOXO3 was reported to positively regulate *ESR1* expression, the expression of a dominant negative form repressing expression of *ESR1* in MCF-7 cells [187], and binding sites were identified in promoters A and B. However, FOXO3 was also reported to suppress the transcriptional activation properties of ERα via protein–protein interactions and to suppress growth of ER+ breast tumor cells [188]. In addition, FOXO3 mediates the cytostatic and cytotoxic properties of breast cancer therapeutics, such as anthracyclins and taxanes, and is down-regulated in MCF-7 cells resistant to epirubicin and paclitaxel [189].

A number of other FOX family members have been reported to regulate ERα function and/or expression. For instance, FOXM1 drives breast cancer cell proliferation under transcriptional control by ERα. It also activates *ESR1* transcription via binding to several sites upstream of promoters A, B and C, possibly in interaction with FOXO3, which bound the same sites and interacted with FOXM1 in co-immunoprecipitation experiments [190]. One site identified in promoter A overlaps with a ChIP peak for FOXA1 [137]. Despite these observations, *FOXM1* is highly expressed in triple negative tumors [191], and in mice inhibits luminal differentiation via inhibition of GATA3 expression [192].

*FOXC1* and *FOXC2* expression patterns are likewise elevated mostly in basal-like tumors [193,194]. FOXC1 repressed *ESR1* expression when overexpressed in three ER+ BC cell lines. ChIP qPCR and pulldown with biotinylated antibodies indicated binding of GATA3 and FOXC1 to overlapping motifs in promoter C and enhancer 4. FOXC1-mediated suppression of *ESR1* was accompanied by exclusion of GATA3 binding, increased H3K9me3, and reduced KDM4B and RNApolII interaction at these sites and upstream of promoter A [120].

Together, these studies suggest a positive role for FOXA1 in *ESR1* up-regulation in ER+ breast cancer cells, with possible cross-talk with several FOX factors.

### 4.3. Estrogen Receptor Alpha

Published ChIP-Seq/ChIP-Exo datasets [19,135,136,137,195] show that ERα binds *ESR1* enhancers 1 to 4; in addition, EREs were detected in the center of the ChIP peaks corresponding to enhancers 1 and 4 (Figure 5). Previous studies reported half-ERE motifs in promoters A [116] and F [114] (Figure 4), although these motifs do not correlate with strong recruitment of ERα at those sites in the ChIP-Exo study. The presence of ERα at distal enhancers is in agreement with reports that ERα is primarily recruited at enhancers rather than promoters of estrogen-modulated genes in breast cancer cell lines [19,135].

Consistent with its recruitment on *ESR1* regulatory regions, ERα exerts feedback regulation on its own expression in a cell context-dependent manner [116,158]. Indeed, *ESR1* mRNA levels are decreased by 17-β-estradiol in MCF-7 and induced in T-47D [196]. The basis for this differential regulation, as well as the reasons dictating the wide variations in *ESR1* expression levels in BC cell lines and tumors, remain unclear. Of note, exogenous expression of ERβ in MCF-7 cells identified genomic targets that for the vast majority overlapped with ERα binding regions [197]. This likely results from the capacity of both receptors to recognize the same binding sites and to heterodimerize; however, divergence in their transactivation regions often result in different effects on transcription, heterodimers having intermediate profiles [198,199]. Whether ERβ can bind *ESR1* regulatory regions and cross-regulate its expression in normal tissue, in which it is expressed to higher levels than in breast tumors, remains to be clarified.

Estradiol treatment also induces ERα turnover in tumor cells, leading to an attenuation of ER signaling [196,200]. The existence of negative feedback mechanisms on ERα levels in the presence of agonists in normal tissue [201] may contribute to the transient nature of the proliferative response to estrogens during the hormonal cycle and may explain in part the lack of correlation between ERα and proliferation markers such as KI67 in the normal human mammary gland (see above). In tumors or in normal tissue of high-risk women [201], bypass of these control mechanisms and ERα overexpression may drive mammary cell proliferation.

## 5. Conclusions

The identification of long-range regulatory elements in *ESR1* by ChIP-Seq analysis together with the mapping of alternative promoters outlines a complex mode of *ESR1* regulation. Transcription factors bind these regulatory elements directly via the recognition of cognate DNA target motifs but may also interact indirectly with other regions via long-range chromatin loops, through direct or co-factor-mediated interactions with other transcription factors. Luminal factors FOXA1 and GATA3 contribute to the positive regulation of the *ESR1* gene, with ERα and possibly ERβ exerting context-dependent feedback regulation. CpG sites methylated in a cell-specific manner are found across the entire *ESR1* gene and likely modulate expression of alternative promoters. Epigenetic modulation via molecules inhibiting histone/DNA methyltransferases or histone deacetylases suggests that *ESR1* expression is controlled in a cell-specific manner by chromatin-modifying/remodeling enzymes potentially recruited to the *ESR1* gene by specific transcription factors [202]. Luminal transcription factors described above likely play an important role in recruiting coactivators such as histone acetyl transferases, and chromatin remodelers such as the SWI-SNF complex. Repressive complexes may be recruited by negative regulators such as FOXC1 or other repressors, including the transcription factor ELF5, shown to repress *ESR1* and a set of ERα-associated genes with promotion of basal-like breast cancer characteristics [203]. Mesenchymal transcription factors have also been observed to repress *ESR1* expression [204,205]. Other regulators not discussed in this review include miRNAs; several are capable of targeting the *ESR1* 3′UTR and some are negatively correlated with *ESR1* in breast cancer [206].

In spite of the accumulated knowledge about regulators of *ESR1* transcription, our understanding of the mechanisms that control ERα expression in normal tissue and how they are altered during breast tumorigenesis requires further investigation. A better dissection of the hierarchical nature of enhancers controlling the *ESR1* gene is required to identify which factors are key to enhancing or silencing *ESR1* expression. Several epigenetic regulators have been proposed to induce *ESR1* expression in triple negative tumors, but whether this will result in therapeutic approaches based on subtype conversion remains unclear. Finally, it is crucially important to better understand reasons for the variable expression patterns of ERα in tumors, the nature of ER-negative cells in heterogeneous ER-positive tumors, the impact of hormonal therapies on these tumors and the mechanisms of the apparent conversion of some ER-positive to ER-negative tumors during cancer progression. The recent availability of single cell and spatially resolved genomic analyses should greatly facilitate the study of intra-tumoral heterogeneity in ERα expression in the near future.

## Data Availability

No new data were created or analyzed in this study. Data sharing is not applicable to this article.
